# Fluorescent nanodiamonds encapsulated by *Cowpea Chlorotic Mottle Virus* (CCMV) proteins for intracellular 3D-trajectory analysis†

**DOI:** 10.1039/d1tb00890k

**Published:** 2021-06-21

**Authors:** Yingke Wu, Shuqin Cao, Md Noor A Alam, Marco Raabe, Sandra Michel-Souzy, Zuyuan Wang, Manfred Wagner, Anna Ermakova, Jeroen J. L. M. Cornelissen, Tanja Weil

**Affiliations:** Max Planck Institute for Polymer Research, Ackermannweg 10 Mainz 55128 Germany weil@mpip-mainz.mpg.de; State Key Laboratory of Oral Diseases, West China Hospital of Stomatology, Sichuan University Chengdu 610041 China; Institute of Inorganic Chemistry I, Ulm University, Albert-Einstein-Allee 11 Ulm 89081 Germany; Department of Molecules & Materials, MESA+Institute for Nanotechnology, University of Twente, P.O. Box 217, 7500 AE Enschede The Netherlands j.j.l.m.cornelissen@utwente.nl; Institute for Measurement and Automation, Division of Sensor Technology and Measurement Systems, Bundeswehr University Munich, Werner-Heisenberg-Weg 39 Neubiberg 85579 Germany; Institute for Physics, Johannes Gutenberg University Mainz, Staudingerweg 7 Mainz 55128 Germany

## Abstract

Long-term tracking of nanoparticles to resolve intracellular structures and motions is essential to elucidate fundamental parameters as well as transport processes within living cells. Fluorescent nanodiamond (ND) emitters provide cell compatibility and very high photostability. However, high stability, biocompatibility, and cellular uptake of these fluorescent NDs under physiological conditions are required for intracellular applications. Herein, highly stable NDs encapsulated with *Cowpea chlorotic mottle virus* capsid proteins (ND-CP) are prepared. A thin capsid protein layer is obtained around the NDs, which imparts reactive groups and high colloidal stability, while retaining the opto-magnetic properties of the coated NDs as well as the secondary structure of CPs adsorbed on the surface of NDs. In addition, the ND-CP shows excellent biocompatibility both *in vitro* and *in vivo*. Long-term 3D trajectories of the ND-CP with fine spatiotemporal resolutions are recorded; their intracellular motions are analyzed by different models, and the diffusion coefficients are calculated. The ND-CP with its brilliant optical properties and stability under physiological conditions provides us with a new tool to advance the understanding of cell biology, *e.g.*, endocytosis, exocytosis, and active transport processes in living cells as well as intracellular dynamic parameters.

## Abstract 2

Fluorescent nanodiamonds are stabilized by a corona of virus capsid proteins. Intracellular diffusion studies were performed suggesting that virus-coated nanodiamonds could be attractive tools for conceptual understanding of cell-virus interactions.

## Graphical abstract

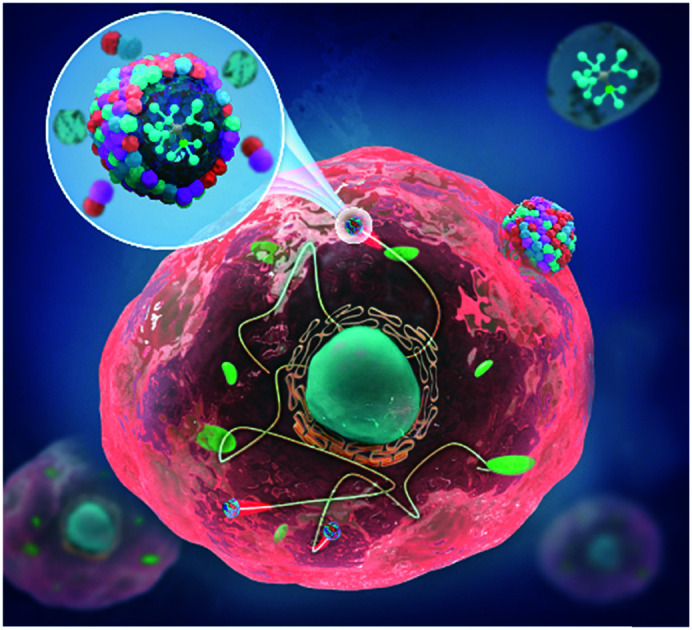

Nanodiamonds (NDs), which consist of an all-carbon sp^3^-scaffold, exhibit both exciting opto-magnetic properties and excellent biocompatibility. Point defects in the lattice of NDs, such as nitrogen-vacancy (NV) centers, provide stable fluorescence without bleaching or blinking, even after several months of continuous irradiation.^1^ In addition, the emission wavelength of NDs is size-independent and tunable from the visible to the near-infrared region by using different defects (*e.g.* SiV, GeV *etc.*).^2^ Hence, NDs are widely used in bioimaging,^3–5^ and drug delivery.^6–10^ In addition, it has been proven that NDs can act as cellular biomarkers for long-term intracellular tracking.^11–13^ Furthermore, NDs containing negatively charged NV centers can also serve as single-spin sensors to detect many essential properties in the biological microenvironment, such as temperature,^14–16^ magnetic fields,^17,18^ electron spins,^19,20^ and strain^21^ with nanoscale resolution.

For most of these applications, appropriate surface functionalization of NDs is required because the poor colloidal stability of unmodified NDs under physiological conditions leads to uncontrolled aggregation in aqueous media.^22^ Surface functionalization, like harsh chemical treatments or air oxidation, improves surface uniformity and provides new binding sites for the attachment of drug molecules, dyes, targeting groups, or antibodies.^23,24^ Furthermore, surface coating is crucial to avoid foreign body interactions of the particles *in vivo*^25^ and allows the nanoparticles to accumulate and remain at the target sites for a prolonged time period. Different surface coatings have been developed for this purpose, such as silica,^26^ hyperbranched polyglycerol (HPG),^27^ poly(l-DOPA),^7^ insulin,^28^ and human serum albumin (HSA), which was modified with polymers,^6^ among others. In addition to synthetic common protein coatings, hybrid virus-like particles have recently gained attention due to their straightforward preparation, high biocompatibility, and unique properties and their application as drug delivery vehicles.^29–31^

Viruses are evolutionary optimized carrier systems. Uptake and diffusion pathways of viruses to their host cells are still barely understood.^32^ However, it is very important to elucidate virus cell interactions and their trafficking inside cells in order to identify new therapeutic interventions or to mimic such processes for improved drug delivery of virus-like nanoparticle transporters. *Cowpea chlorotic mottle virus* (CCMV) has gained great interest in recent years as tools in nanotechnology as well as for the development of targeted drug delivery vehicles due to their perfectly defined structure, high stability, good biocompatibility, homogeneity, self-assembly, and low toxicity.^33^ The size of the native CCMV is about 30 nm in diameter and can be disassembled into capsid proteins (CP). The genome can be removed by centrifugation under high salt concentrations at neutral pH.^34^ The thus obtained CPs reassemble into either empty capsids at lower pH or virus-like particles (VLPs) at neutral pH using templates such as negatively charged inorganic nanoparticles,^35,36^ negatively charged polymers,^37^ enzymes,^38^ and organic aggregates^39^ among others. Furthermore, by controlling the pH and ionic strength of the assembly buffer, CCMV can be reassembled into a variety of geometries such as tubes, multilayered structures, and dumbbells.^40–43^ Despite their broad applications,^44,45^ CCMV cell uptake and diffusion inside cells is still barely understood.

Here, we report the preparation and characterization of fluorescent nanodiamonds encapsulated within *cowpea chlorotic mottle virus* capsid proteins (ND-CP) as model system for fluorescence imaging and single particle tracking applications inside living cells. We believe that our strategy could also be applicable to other viruses, which could give new insights into their intracellular pathways, which would be of fundamental interest as well as for the design of improved drug delivery systems.

A schematic outline of the preparation of ND-CP is displayed in Fig. 1. Firstly, CPs were isolated from the *CCMV* and stored in a capsid storage buffer (50 mM NaOAc, 500 mM NaCl, pH 5) according to our previous work.^36^ Before encapsulation of the NDs, the CPs were transferred into a coat protein buffer (Tris buffer (50 mM Tris, 50 mM NaCl, 10 mM KCl, pH 7.2), or PBS buffer (PBS, 150 mM NaCl, pH 7.2)). Then NDs in a polyvinylpyrrolidone (PVP) solution were added to the CPs and the mixture was stirred at 4 °C overnight.

**Fig. 1:**
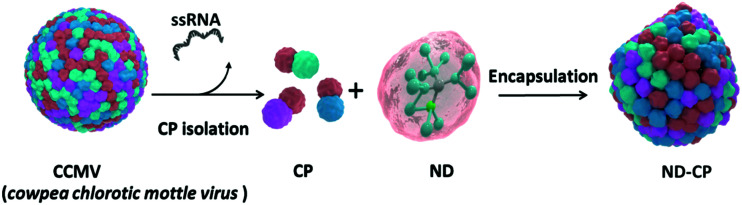
Schematic illustration of the preparation of *cowpea chlorotic mottle virus* capsid protein encapsulated fluorescent nanodiamonds (ND-CP).

Free CPs and encapsulated ND-CPs were separated and removed by fast protein liquid chromatography (FPLC) equipped with a UV-vis detector. As shown in Fig. 2A, the NDs coated with CP both in PBS buffer and Tris buffer eluted at a lower volume (*V* ≈ 8 mL) than the native CCMV virus (*V* ≈ 9.5 mL, from our previous results),^35^ suggesting that NDs were successfully coated. The successful coating of NDs with CPs was also confirmed by sodium dodecyl sulfate-polyacrylamide gel electrophoresis (SDS-PAGE) (Fig. S2, ESI†), which shows that the band of purified ND-CP appeared at the same position as native CCMV. To test whether the CPs retained their native structure after adsorption to the ND surface, the secondary structure was investigated by circular dichroism (CD) spectroscopy. As shown in Fig. 2B, only native CP and ND-CP showed a signal in CD, and both spectra were very similar, indicating that the native structure of CP was not affected. Subsequently, the hydrodynamic sizes of the ND-CP were characterized by dynamic light scattering (DLS) (Fig. 2C). The average hydrodynamic diameters changed from 35 ± 2 nm in water before coating to 46 ± 3 nm for NDs in Tris buffer containing PVP, and 52 ± 5 nm for ND-CP in Tris buffer, respectively. Furthermore, ND-CP showed a monomodal distribution in DLS. Uncoated NDs and ND-CP were characterized in terms of their distribution, shape, and morphology by transmission electron microscopy (TEM). Whereas bare NDs were prone to significant aggregation and displayed a heterogeneous distribution (Fig. 2D), non-aggregating, homogeneous single particles were observed for ND-CP (Fig. 2E). The histogram analysis of TEM images of ND and ND-CP showed a size increase from 15.9 ± 10.6 nm to 23.2 ± 11.2 nm, respectively (Fig. S3, ESI†). From the TEM images (Fig. S4, ESI†), we determined a shell thickness of 3.03 ± 0.96 nm (*n* = 36) of ND-CP, which corresponds nicely to the thickness of the *CCMV* capsid, as also observed by Cheng Zeng *et al*.^46^ They found that the protein shell retains its sub unit such as pentamers and hexamers and preserves the intrinsic interfacial interactions when coated around gold nanorods. The diameter of ND-CP is different in DLS and TEM, because the TEM considers the ND diameter extracted from the images, whereas DLS takes into account the solvated shell with CP and bound water molecules. To further assess the structure of the ND-CP, the liquid mode atomic force microscopy (AFM; Fig. S5, ESI†) was applied. The topographic image of ND-CP showed a narrow size distribution with no significant aggregation. The height-sensor images recorded by AFM were converted into deformation images using the NanoScope Analysis 1.8 software to visualize the CP coating. In addition to the height profile images, other nanomechanical properties were simultaneously recorded. In particular, the deformation of the sample caused by the probe was analyzed to obtain in-depth information on the structure of the coated NDs. As the ND core is much harder than the CP shell, the deformation of the CP shell under the same stress conditions could be detected with a high intensity. The deformation image revealed clearly that all ND-CP possessed a core–shell structure.

**Fig. 2:**
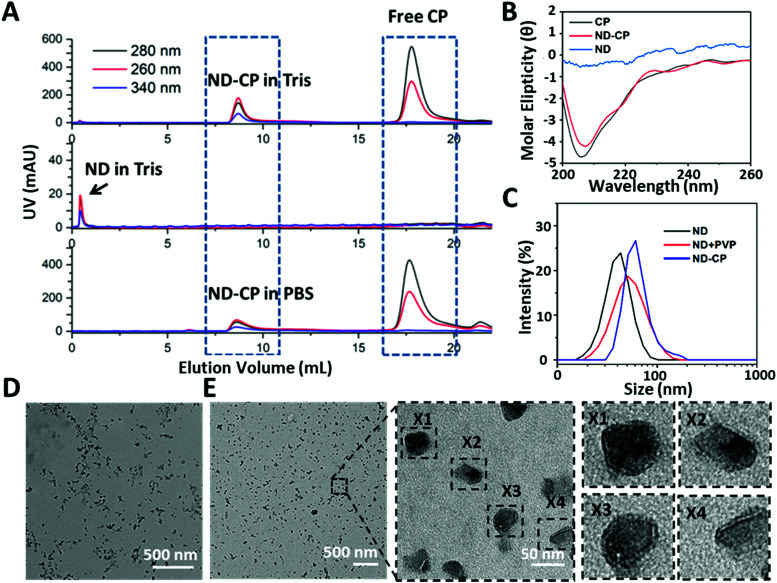
(A) FPLC of ND and ND-CP in Tris buffer (50 mM Tris, 50 mM NaCl, 10 mM KCl, pH 7.2), ND-CP in PBS buffer (PBS, 150 mM NaCl, pH 7.2); (B) CD spectra of ND, CP and ND-CP; (C) DLS diagram of uncoated NDs in water, ND + PVP and ND-CP in Tris buffer; (D) TEM image NDs (scale bar: 500 nm); (E) TEM images of ND-CP at different magnifications (negatively stained with 4% uranyl acetate).

To prove that the optical properties of NDs were retained, the influence of the CP shell on the charge state of the NV centers in NDs was investigated (Fig. 3A). Spectra measurements were performed on a custom-built confocal microscope with an excitation laser at *λ* = 532 nm and 100 μW power in front of the objective (oil, NA = 1.35) (Fig. S1, ESI†). The spectra of ND-CP revealed that the CP on the surface of NDs did not affect the fluorescence, and the zero phonon lines of NV^−^ centers were clearly visible without any shift or background noise. NV^−^ centers in NDs are very sensitive to the surface states and their charge state can switch to neutral or positive, which is optically nonactive. These results demonstrate that the CP coating did not affect the charge properties of NV centers. These remained in the optical and spin active state (NV^−^), which is essential for applications of ND-CP in bioimaging, tracking, and nanoscale sensing. The biocompatibility of ND-CP was investigated using HeLa cell line as well as in a chorioallantoic membrane (HET-CAM) *ex vivo* model.^47^ As depicted in Fig. 3B, the ND-CP showed low cytotoxicity upon treating cells at high concentrations of up to 200 μg mL^−1^. It is worth noting that the HET-CAM method represents a potential alternative to animal experiments to assess toxicity and is widely applied to replace animal experiments.^47^ In our HET-CAM assay, we observed hemorrhage from blood vessels that started within 2–4 seconds after applying 1% sodium dodecyl sulfate (SDS) as positive control. Lysis occurred after 27 seconds, and we observed coagulation within 24 hours. The negative control (phosphate buffered saline) and two concentrations of ND-CP (50 μg mL^−1^, 100 μg mL^−1^) did not show any irritation (such as hemorrhage, vascular lysis, or coagulation) within 5 minutes to 24 hours after application (Fig. S6, ESI†). A summary of the results is shown in Table S2 (ESI†). These *in ovo* results verify the high biocompatibility of ND-CP.

**Fig. 3:**
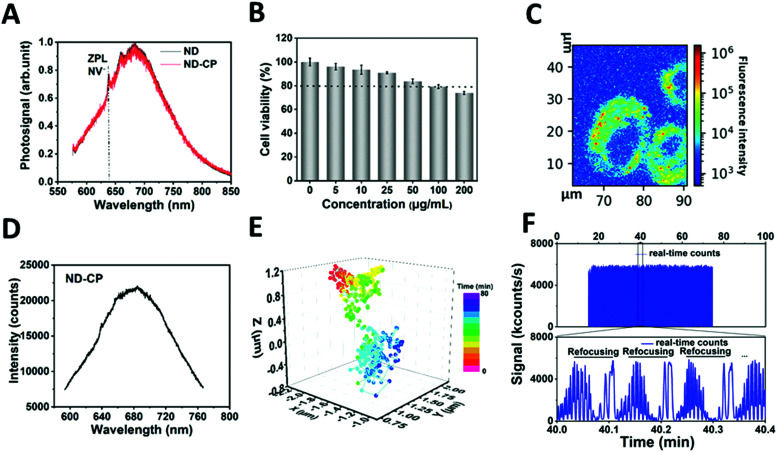
(A) Normalized emission spectra (ex. 532 nm) of ND and ND-CP. NV^−^ zero phonon lines are visible in both spectra. (B) Cell viability of ND-CP in HeLa cells. (C) *X*–*Y* axis confocal microscopy images of ND-CP taken up into HeLa cells at 20 μg mL^−1^ for 4 hours incubation. (D) Emission spectra (ex. *λ* = 532 nm) of ND-CP in HeLa cell. (E) The trajectory of tracked ND-CP spot 1 in the intracellular space of HeLa cell. (F) Real-time counts of fluorescence of the tracked ND-CP spot 1 with continuous refocusing (upper panel) and regional enlarged view showing three refocusing steps (lower panel).

To track the motion of the ND-CP inside living cells, HeLa lung cancer cells were incubated with 20 μg mL^−1^ ND-CP for 4 hours. The measurements were performed on a custom-built confocal microscope with an excitation laser at 532 nm (Fig. S1, ESI†). As depicted in Fig. 3C, ND-CPs were efficiently taken up by HeLa cells at concentrations of 20 μg mL^−1^. To track ND-CP nanoparticles, the spectrum of the selected spots inside living cells was first measured to confirm the presence of ND-CP in the focus volume (Fig. 3D). Then, the fluorescence intensities of the tracked ND-CPs were recorded simultaneously to ensure that the same ND-CP was being tracked (Fig. 3F). A representative trajectory of single particles is shown in Fig. 3E for 43 minutes and two other trajectories are depicted in Fig. S7 (ESI†).

The high photostability of NDs provides the opportunity to study intracellular dynamic parameters of ND-CP inside living cells. We performed a set of proof of principle experiments by recording 3D trajectories of three different ND-CP spots. Trajectory 1 was observed for 43 minutes containing 520 points, whereas Trajectories 2 and 3 were recorded for 7 minutes with 88 and 81 points, respectively. For trajectories 2 and 3, long-time trajectory measurements did not succeed due to interference from other ND-CPs that could not be distinguished during the refocusing process. With increasing tracking time, the probability of interference from nearby ND-CPs increases as well, thus limiting the maximum tracking time for individual ND-CPs. The obtained data points allowed calculating the mean square displacement (MSD), which is a measure of the deviation of the position of ND-CP nanoparticles with respect to a reference position over time. The results for each ND-CP spot are presented in Fig. 4A (see MSD analysis for calculations details in ESI†). Subsequently, the diffusion behavior of the ND-CPs was obtained by fitting the MSD data with a power-law (eqn (S2), ESI†), where the power index *α* reflects the diffusion behavior of the tracked nanoparticle with *α* < 1, *α* = 1, and *α* > 1 to identify the dynamic movements of ND-CPs inside cells.^48,49^ Within the complex and highly heterogeneous, crowded environment in the cell's cytoplasm, normal diffusion, anomalous diffusion, confined diffusion or directed motion of nanoparticles may occur.^50^ In normal diffusion, nanoparticles diffuse completely unrestricted, whereas directed motion represents an active process that has been observed when small vesicles are transported by molecular machines along microtubules.^51,52^ Confined diffusion has been observed for trapped particles or particles whose free diffusion is confined by cytoskeletal elements.^53^ However, the origin of anomalous diffusion is commonly traced back to the macromolecular crowding in the interior of cells, but its precise nature is still under discussion.^50,54^

**Fig. 4:**
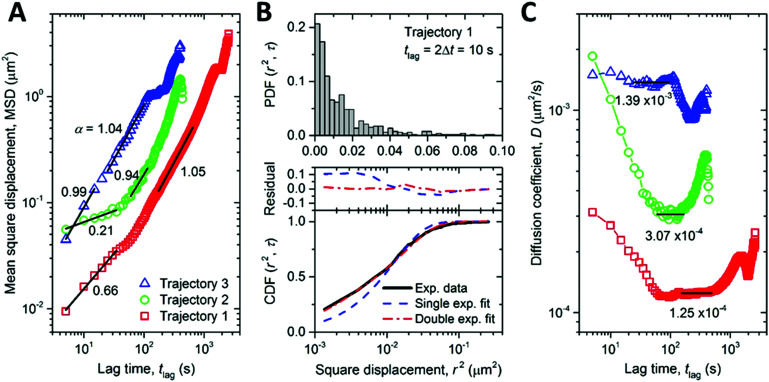
(A) Mean square displacement (MSD) *vs.* lag time (*t*_lag_) for three independent trajectories. The segments of the MSD profiles at short and intermediate lag times are analyzed using a power law relation. The power indices are indicated. (B) Probability density function (PDF, upper panel, note the linear scale of the *x* axis) and cumulative density function (CDF, lower panel) distributions of the square displacements at *t*_lag_ = 2Δ*t* = 10 s. The CDF distribution is fitted with single and double exponential functions. The residuals of the fitting are shown on the top of the lower panel. (C) Time-dependent diffusion coefficient (*D*) of the three trajectories (see (*A*) for the legends). The nominal *D* values are calculated as the average values at intermediate lag times, as indicated by the black horizontal lines. In these ranges of the lag times, the power indices in (*A*) are close to one, implying normal diffusion.

According to Saxton,^55^ we focused on data with *t*_lag_ < *t*_total_/4, where *t*_total_ is the total time of the trajectory. Our experiments showed a confined diffusion at the short lag time, which was also observed for single-walled-carbon-nanotube (SWNT)-labeled kinesins in COS-7 cells before.^56^ Previously, this behavior was allocated to the existence of mechanical obstacles such as microtubule-microtubule intersections that exist in the complex cytoskeletal network of cells.^56,57^ We have distinguished normal and anomalous diffusion of ND-CPs by determining the cumulative distribution function (CDF) of the square displacements at a given *t*_lag_.^48,58^ To provide the CDF computation, the probability distribution function (PDF) of the square displacements was first calculated. The PDF and CDF were analyzed only for trajectory 1 because of the largest number of points, as shown in Fig. 4B. The CDF data were fitted with single and double exponential functions, where the double function has fast and slow mobility components. The double fit has a much smaller residual than the single exponential fit. The ratio between the fast and slow mobility parts in the double fit confirms that the diffusion behavior of the ND-CP in HeLa cells is indeed a combination of normal (*i.e.*, MSD ∝ *t*_lag_) and anomalous (*i.e.*, MSD ∝ *t*_lag_^*α*^, with *α* deviating from one) diffusion. The diffusion coefficient (*D*_0_) calculated from the single exponential fit is equal to 2.09 × 10^−4^ μm^2^ s^−1^. The double exponential fit gives two diffusion coefficients *D*_1_ = 2.95 × 10^−5^ μm^2^ s^−1^ and *D*_2_ = 2.93 × 10^−4^ μm^2^ s^−1^. We also calculated the lag-time-dependent diffusion coefficient of the ND-CP in HeLa cell from the MSD data for each of the three recorded trajectories (for more details see ESI†). We obtained the diffusion coefficients of 1.25 × 10^−4^ μm^2^ s^−1^, 3.07 × 10^−4^ μm^2^ s^−1^, and 1.39 × 10^−3^ μm^2^ s^−1^ for trajectory 1, 2, and 3, respectively (Fig. 4C). They are comparable to the values obtained from the single and double exponential fits of the CDF data. The nominal diffusion coefficient of the ND-CP in the HeLa cell was then determined to be the average of these three values, that is, 6.07 × 10^−4^ μm^2^ s^−1^.

The trajectories in Fig. 4A exhibit anomalous diffusion (*α* < 1) at short time and normal diffusion (*α* ≈ 1) at long time, which, according to Saxton and Jacobson,^59^ could be attributed to random point obstacles. Interestingly, the recorded intracellular trajectories of ND-CPs are in line with previous reports on the diffusion of adeno-associated virions in the cytoplasm of a living HeLa cell, where intracellular trajectories have also been classified as anomalous diffusion and normal diffusion.^59,60^ The interactions between the viruses and obstacles could involve nonspecific interacting (*e.g.*, touching), short-term binding/releasing, long-term binding, and penetration events. It has been shown that in the infection pathway the adeno-associated viruses experienced a mean of 4.4 repetitive touching events per virus and a penetration efficiency of 13%.^61^ This suggests that the majority of the interactions are not strong interactions that would cause stopping or binding of the viruses; instead, they only hinder the free diffusion of the viruses, leading to anomalous diffusion. We speculate that this mechanism could also be the origin of the anomalous diffusion of ND-CPs in our study. Therefore, ND-CPs consisting of a fluorescent nanodiamond core and capsid protein corona could represent valuable tools to elucidate the infection pathway of the virions over the cytoplasm.

## Conclusions

In conclusion, we have developed a virus-inspired NDs hybrid system consisting of a negatively charged fluorescent nanodiamond core stabilized by a corona of virus capsid proteins. ND-CP showed excellent stability in PBS and Tris buffer. Both the NDs and CPs retained their unique features, such as photophysical properties and 3D structure facilitating cellular uptake. Furthermore, they showed good biocompatibility up to 100 μg mL^−1^*in vitro* and *in vivo*, which is essential for bioapplications, such as bioimaging and intracellular trajectory analysis. The estimation of intracellular motions of ND-CP revealed confined diffusion at the beginning, which then changed to normal diffusion (*i.e.*, MSD ∝ *t*_lag_), which has also been observed for adenovirus-like particles. We believe that ND-CP represent a useful tool to study the intracellular motions over long time periods and with spatiotemporal details in living cells. In combination with optically detected magnetic resonance technique,^62^ one could also envision simultaneous elucidation of intercellular magneto-, electro-, and temperature-information at the nanoscale, which will allow acquiring quantitative information on the intracellular pathways of virions. The trajectory analysis of the ND-CP have the potential to enhance the conceptual understanding of cell–virus interactions, host adaption, and immune regulation, and elucidate the interactions between eukaryotes and viruses during infection.

## Conflicts of interest

The authors declare no competing financial interest.

## Supplementary Material

TB-009-D1TB00890K-s001

## References

[cit1] Miller B. S., Bezinge L., Gliddon H. D., Huang D., Dold G., Gray E. R., Heaney J., Dobson P. J., Nastouli E., Morton J. J. (2020). Nature.

[cit2] Xiao J., Liu P., Li L., Yang G. (2015). J. Phys. Chem. C.

[cit3] Han S., Raabe M., Hodgson L., Mantell J., Verkade P., Lasser T., Landfester K., Weil T., Lieberwirth I. (2019). Nano Lett..

[cit4] Torelli M. D., Nunn N. A., Shenderova O. A. (2019). Small.

[cit5] Jung H. S., Cho K. J., Seol Y., Takagi Y., Dittmore A., Roche P. A., Neuman K. C. (2018). Adv. Funct. Mater..

[cit6] Wu Y., Ermakova A., Liu W., Pramanik G., Vu T. M., Kurz A., McGuinness L., Naydenov B., Hafner S., Reuter R. (2015). Adv. Funct. Mater..

[cit7] Harvey S., Raabe M., Ermakova A., Wu Y., Zapata T., Chen C., Lu H., Jelezko F., Ng D. Y., Weil T. (2019). Adv. Ther..

[cit8] Zhang Y., Cui Z., Kong H., Xia K., Pan L., Li J., Sun Y., Shi J., Wang L., Zhu Y. (2016). Adv. Mater..

[cit9] Cui Z., Zhang Y., Xia K., Yan Q., Kong H., Zhang J., Zuo X., Shi J., Wang L., Zhu Y. (2018). Nat. Commun..

[cit10] Ryu T. K., Baek S. W., Kang R. H., Choi S. W. (2016). Adv. Funct. Mater..

[cit11] Fu C.-C., Lee H.-Y., Chen K., Lim T.-S., Wu H.-Y., Lin P.-K., Wei P.-K., Tsao P.-H., Chang H.-C., Fann W. (2007). Proc. Natl. Acad. Sci. U. S. A..

[cit12] Chang Y.-R., Lee H.-Y., Chen K., Chang C.-C., Tsai D.-S., Fu C.-C., Lim T.-S., Tzeng Y.-K., Fang C.-Y., Han C.-C. (2008). Nat. Nanotechnol..

[cit13] Zhang B., Li Y., Fang C. Y., Chang C. C., Chen C. S., Chen Y. Y., Chang H. C. (2009). Small.

[cit14] Neumann P., Jakobi I., Dolde F., Burk C., Reuter R., Waldherr G., Honert J., Wolf T., Brunner A., Shim J. H. (2013). Nano Lett..

[cit15] Simpson D. A., Morrisroe E., McCoey J. M., Lombard A. H., Mendis D. C., Treussart F., Hall L. T., Petrou S., Hollenberg L. C. (2017). ACS Nano.

[cit16] Sekiguchi T., Sotoma S., Harada Y. (2018). Biophys. Physicobiol..

[cit17] Ermakova A., Pramanik G., Cai J.-M., Algara-Siller G., Kaiser U., Weil T., Tzeng Y.-K., Chang H.-C., McGuinness L., Plenio M. B. (2013). Nano Lett..

[cit18] Zhang T., Liu G.-Q., Leong W.-H., Liu C.-F., Kwok M.-H., Ngai T., Liu R.-B., Li Q. (2018). Nat. Commun..

[cit19] Horowitz V. R., Alemán B. J., Christle D. J., Cleland A. N., Awschalom D. D. (2012). Proc. Natl. Acad. Sci. U. S. A..

[cit20] Akiel R. D., Zhang X., Abeywardana C., Stepanov V., Qin P. Z., Takahashi S. (2016). J. Phys. Chem. B.

[cit21] Xia K., Liu C.-F., Leong W.-H., Kwok M.-H., Yang Z.-Y., Feng X., Liu R.-B., Li Q. (2019). Nat. Commun..

[cit22] van der Laan K., Hasani M., Zheng T., Schirhagl R. (2018). Small.

[cit23] Krueger A., Lang D. (2012). Adv. Funct. Mater..

[cit24] Krueger A., Nanodiamonds, Elsevier, 2017, pp. 183–242

[cit25] Zhao L., Xu Y.-H., Akasaka T., Abe S., Komatsu N., Watari F., Chen X. (2014). Biomaterials.

[cit26] Bumb A., Sarkar S. K., Billington N., Brechbiel M. W., Neuman K. C. (2013). J. Am. Chem. Soc..

[cit27] Zhao L., Takimoto T., Ito M., Kitagawa N., Kimura T., Komatsu N. (2011). Angew. Chem., Int. Ed..

[cit28] Shimkunas R. A., Robinson E., Lam R., Lu S., Xu X., Zhang X.-Q., Huang H., Osawa E., Ho D. (2009). Biomaterials.

[cit29] Tyler M., Tumban E., Peabody D. S., Chackerian B. (2014). Biotechnol. Bioeng..

[cit30] Zhu W., Fang T., Zhang W., Liang A., Zhang H., Zhang Z.-P., Zhang X.-E., Li F. (2021). Nanoscale.

[cit31] Wang J., Fang T., Li M., Zhang W., Zhang Z.-P., Zhang X.-E., Li F. (2018). J. Mater. Chem. B.

[cit32] Ruthardt N., Lamb D. C., Bräuchle C. (2011). Mol. Ther..

[cit33] Hema M., Vardhan G. V., Savithri H. and Murthy M., in Recent developments in applied microbiology and biochemistry, ed. V. Viswanath, Elsevier, Amsterdam, 2019, ch. 6, pp. 47–60

[cit34] Douglas T., Young M. (1998). Nature.

[cit35] Liu A., de Ruiter M. V., Zhu W., Maassen S. J., Yang L., Cornelissen J. J. (2018). Adv. Funct. Mater..

[cit36] Liu A., Verwegen M., de Ruiter M. V., Maassen S. J., Traulsen C. H.-H., Cornelissen J. J. (2016). J. Phys. Chem. B.

[cit37] Minten I. J., Ma Y., Hempenius M. A., Vancso G. J., Nolte R. J., Cornelissen J. J. (2009). Org. Biomol. Chem..

[cit38] Schoonen L., Nolte R. J., van Hest J. C. (2016). Nanoscale.

[cit39] Sinn S., Yang L., Biedermann F., Wang D., Kübel C., Cornelissen J. J., De Cola L. (2018). J. Am. Chem. Soc..

[cit40] Bancroft J., Hiebert E., Bracker C. (1969). Virology.

[cit41] Lavelle L., Gingery M., Phillips M., Gelbart W., Knobler C., Cadena-Nava R., Vega-Acosta J., Pinedo-Torres L., Ruiz-Garcia J. (2009). J. Phys. Chem. B.

[cit42] Speir J. A., Munshi S., Wang G., Baker T. S., Johnson J. E. (1995). Structure.

[cit43] Douglas T., Strable E., Willits D., Aitouchen A., Libera M., Young M. (2002). Adv. Mater..

[cit44] Chan S. K., Du P., Ignacio C., Mehta S., Newton I. G., Steinmetz N. F. (2021). ACS Nano.

[cit45] Cai H., Shukla S., Steinmetz N. F. (2020). Adv. Funct. Mater..

[cit46] Zeng C., Rodriguez Lázaro G., Tsvetkova I. B., Hagan M. F., Dragnea B. (2018). ACS Nano.

[cit47] Winter G., Koch A. B., Löffler J., Lindén M., Solbach C., Abaei A., Li H., Glatting G., Beer A. J., Rasche V. (2020). Cancers.

[cit48] Weigel A. V., Simon B., Tamkun M. M., Krapf D. (2011). Proc. Natl. Acad. Sci. U. S. A..

[cit49] Gal N., Lechtman-Goldstein D., Weihs D. (2013). Rheol. Acta.

[cit50] Wagner T., Kroll A., Haramagatti C. R., Lipinski H.-G., Wiemann M. (2017). PLoS One.

[cit51] Ruan G., Agrawal A., Marcus A. I., Nie S. (2007). J. Am. Chem. Soc..

[cit52] Bannunah A. M., Vllasaliu D., Lord J., Stolnik S. (2014). Mol. Pharmaceutics.

[cit53] Monnier N., Guo S.-M., Mori M., He J., Lénárt P., Bathe M. (2012). Biophys. J..

[cit54] Höfling F., Franosch T. (2013). Rep. Prog. Phys..

[cit55] Saxton M. J. (1997). Biophys. J..

[cit56] Fakhri N., Wessel A. D., Willms C., Pasquali M., Klopfenstein D. R., MacKintosh F. C., Schmidt C. F. (2014). Science.

[cit57] Bálint Š., Vilanova I. V., Álvarez Á. S., Lakadamyali M. (2013). Proc. Natl. Acad. Sci. U. S. A..

[cit58] Schütz G. J., Schindler H., Schmidt T. (1997). Biophys. J..

[cit59] Saxton M. J., Jacobson K. (1997). Annu. Rev. Biophys. Biomol. Struct..

[cit60] Itto Y., 2018, peprint, arXiv:1803.03097

[cit61] Seisenberger G., Ried M. U., Endress T., Büning H., Hallek M., Bräuchle C. (2001). Science.

[cit62] Schirhagl R., Chang K., Loretz M., Degen C. L. (2014). Annu. Rev. Phys. Chem..

