# Supplementary material for: Fluorescent nanodiamonds encapsulated by Cowpea Chlorotic Mottle Virus (CCMV) proteins for intracellular 3D-trajectory analysis
Source: J Mater Chem B. 2021 Jun 21;9(28):5621–7. doi: 10.1039/d1tb00890k (PMC8292973; doi:10.1039/d1tb00890k)
Supplement: TB-009-D1TB00890K-s001 [file TB-009-D1TB00890K-s001.pdf]

Electronic Supplementary Material (ESI) for Journal of Materials Chemistry B.
This journal is © The Royal Society of Chemistry 2021

Supplementary Information

Fluorescent Nanodiamonds Encapsulated by *Cowpea Chlorotic Mottle Virus* (CCMV) Proteins for Intracellular 3D-Trajectory Analysis

Yingke Wu†, Shuqin Cao†, Md Noor A Alam, Marco Raabe, Sandra Michel-Souzy, Zuyuan Wang, Manfred Wagner, Anna Ermakova, Jeroen J.L.M. Cornelissen*, Tanja Weil*

CONTENTS:
1. Experimental section
   Materials
   Methods
2. Tables and Figures
3. References

1. Experimental section

Materials: Nanodiamonds with 35 nm average diameter were purchased from FND Biotech (Taiwan), polyvinylpyrrolidone (MW: 10 kDa) was purchased from Sigma-Aldrich, Quartz glass cuvette were purchased from Hellma-analytics, Quartz SUPRASIL (QS). Amicon Ultra centrifuge filter with 10K MWCO from Pall MicrosepTM Advance Centrifuge Device with Omega Membrane were used for particle preparation.

Dulbecco's Modified Eagle's Medium (DMEM, 1x), Dulbecco's phosphate-buffered saline (DPBS, 1x), fetal bovine serum (FBS), Penicillin Streptomycin (Pen Strep) were purchased from gibco. All solvents and chemicals were purchased from commercial sources and were used without further purification

Preparation of CCMV

Pots are filled with soil and the Cowpea beans are planted in the soil. The plants are allowed to grow for ten days and watered regularly. At this point primary leaves are present. The leaves are dusted with carborundum. The inoculation solution is prepared with a purified virus. In both cases a mixture of sap from ground leaves, water and purified virus solution (0.5 mg virus) is prepared. The leaves are inoculated by smoothly rubbing. By using a household sprayer, the plants are immediately sprayed with water after inoculation to prevent leaf dehydration[1]. Seven days after inoculation, the leaves are harvested by cutting them from the stem. The plant material is cut into pieces and blended in cold homogenization buffer (0.2 M NaAc, 0.01 M ascorbic acid, 0.01 M Na2EDTA)[2]. Two layers of cheesecloth are boiled in water containing EDTA and rinsed with MilliQ water. The homogenate is filtrated to remove the larger plant debris. The homogenate is kept at

T=4 °C for an hour to allow the proteins to precipitate.[3] The homogenate is subjected to low-speed centrifugation to precipitate the leaf tissue. The pellet is discarded and the supernatant is added to 10 % (w/v) solid PEG (MW=6000 g/mol). The mixture is stirred for 1 h at T=4 °C. The precipitate is pelleted by low-speed centrifugation. The supernatant is discarded and the bottles are drip-dried thoroughly to remove the PEG solution (Hebert, T. T., Precipitation of plant viruses by polyethylene glycol. Phytopathology 1963, 53, 362.) The pellet is suspended in cold virus buffer (0.1 M NaAc, 1 mM Na2EDTA, 1 mM NaN3) with the help of a glass stick or a pipette. The resuspension is cleared of undissolved material by low-speed centrifugation.

The pellet is discarded and the supernatant is mixed with cesium chloride. Once completely dissolved, the mixture is subjected to a density gradient centrifugation. The obtained solution is dialyzed against virus buffer at T= 4°C, and then stored at T=4 °C. The presence and purity is checked by SDS-PAGE and FPLC. Typical yields of CCMV are 200–300 mg per kg of cowpea tissue. During the whole process, the virus solution is kept cold either in an ice bath or in the cold room (T= 4°C).

Preparation of CP

RNA in a suspension of CCMV (1 mL 10 mg/mL) is precipitated by dialysis against RNA buffer (0.05 M Tris-HCl, 0.5 M $CaCl_2$, 1 mM DTT) at 4 °C. The white precipitate containing RNA is centrifuged down. The supernatant is removed and the pellet discarded. The supernatant is afterwards dialyzed against cleaning buffer (0.05 M Tris-HCl, 0.5 NaCl, 1 mM DTT). The dissociated protein without RNA is obtained and associated into spherical particles by dialysis against capsid storage buffer (0.05 M NaAc, 1 M NaCl, 1mM NaN_3).[4]

Preparation of ND-CP

In a typical experiment, NDs solution (400 µL, 0.2 mg/mL; H_2O) is added to a solution of CCMV coat protein (100 µL, 15 mg/mL; pH 7.2; 250 mM Tris, 500 mM NaCl) and allowed to incubate overnight at 4 ^{0}C. The reaction mixture is subsequently resulting CP-NDs are purified using preparative FPLC.

Purification of ND-CP by fast protein liquid chromatography (FPLC)

FPLC analysis were performed on a GE Healthcare ÄKTApurifierTM system equipped with a Superose 610/300 GL column from GE Healthcare and a fractionating device. Injection of 500 µL pre-filtered samples which are injected on a 24 mL superpose-6 column. Compound elution is monitored using a UV-vis spectrometer at 260 nm, 280 nm. Fractionation are collected separately

Sodium dodecyl sulfate–polyacrylamide gel electrophoresis (SDS-PAGE)

SDS-PAGE samples are prepared by mixing 10 µL of sample with 9 µL of sample buffer (125 mM Tris-HCl, 20% (v/v) glycerol, 5% (w/v) sodium dodecyl sulfate, 0.02% (w/v) bromophenol blue, pH 6.8) and 1 µL 2-mercaptoethanol. The mixture was heated at 99 °C for 5 minutes to denature the protein, after which the mixture was used to fill the wells of 4-15% stain free pre-cast poly acryl amide gels (Bio-Rad). Precision Plus Protein™ Unstained Protein Standard was added in a separate well. Electrophoreses was conducted at 100 V for 5 minutes followed by 200Vfor approximately 20 minutes. Gels where activated with UV for 5 minutes on a stain-free enabled UV transilluminator and imaged

with a Gel Doc™ EZ system with Image Lab software (Bio-Rad).

Dynamical light scattering (DLS)

DLS analysis was performed using a Nanotrac (Anaspec) instrument. Microtrac FLEX Operating software was used at 25°C with laser wavelength of 780 nm and a scattering angle of 90°. The observed size and standard deviation of the nanoparticles were calculated by taking an average of 5 measurements.

Transmission Electron Microscopy (TEM)

4 µL 0.1 mg/mL solution of ND-CPs in MilliQ was placed onto an oxygen treated carbon coated copper grid. After 10 minutes the solution was removed using a filter paper and grids were stained with uranyl acetate 4% for 1.5 minutes. The grids were washed three times with MilliQ water and dried before measuring. A Jeol 1400 transmissions electron microscope was used to obtain bright field images. And Image J software was used to process the data.

Atomic Force Microscopy (AFM)

Atomic force microscopy was conducted in liquid state with a Bruker Dimension FastScan BioTM atomic force microscope, which was operated in PeakForce mode. AFM probes with a nominal spring constant of 0.25 N m-1 (FastScan-D, Bruker) were used. The samples were diluted with MilliQ water to a concentration of 0.05 mg mL-1. Sample solution (30 µL) was added onto a freshly cleaved mica substrate (circular, 15 mm) and incubated for at least 10 minutes to allow deposition of the structures. Remaining solution

was removed and 300 μL MilliQ water was applied onto the mica surface, forming a droplet for measuring in liquid. Samples were scanned with scan rates between 1 and 2 Hz and scan sizes between 0.5 and 2 μm. Images were processed with NanoScope Analysis 1.8.

Cytotoxicity measurements

HeLa cells were seeded with DMEM (contains 10% FBS, 1% MEM NEAA, and 1% PenStrep) on a 96 well half-area flat bottom microplate (50 μL/well, 1.3×10^5 cells/mL), the plate was then incubated at 37 °C, 5% CO_2 overnight. Next day, different concentrations of ND-CP samples were added to the cells and incubated again for overnight. Next day after removing the ND-CP containing medium and washing each well three times with DPBS, luminescent cell viability assay (CellTiter-Glo) was added to the cells as instructed by the manufacturer's protocol. To measure the luminescence, a Promega GloMax plate reader was utilized.

Hen's Egg Test on the Chorioallantoic Membrane (HET-CAM)

Fertile eggs were purchased from LSL Rhein-Main Geflügelvermehrungsbetriebe GmbH & Co.KG, Germany. The eggs were wipe-cleaned carefully, then incubated at 37 °C for 3 days. The pointy end of the eggs were placed downward during the incubation. From the first day of incubation, we started to count the so called Embryo development day (EDD). On the EDD 3, using a syringe and needles- about 6 mL egg albumin was removed from each egg. Afterward, a small portion of the eggshell was carefully cut with a surgical scissor and removed from the wide end of the eggs. By employing a thin parafilm tape, the

open area was sealed again. The eggs were then incubated again for 7 more days till EDD 10. The eggs were inspected frequently (at least once every 24 hours) to remove any nonviable egg.

On the EDD 10, eggs with healthy embryo were separated and treated with ND-CP, positive and negative controls (1% SDS and PBS). During the material dropping, eggs were always placed near a light bulb to simulate the incubator temperature. The samples were dropped directly on the chorioallantoic membrane of three eggs, 10 μL of each sample. Before adding the materials and after 5 minutes of application of the samples, high resolution photographs were taken. We also recorded videos continuously for 5 minutes during the application of ND-CP, SDS, and PBS on CAM of each eggs to detect any exhibition of hemorrhage, coagulation, or vascular lysis. After adding the materials- eggs were placed in the incubator again. Next day, after about 24 hours, photograph was taken of the material application sites.

We used the Hen's Egg Test on the Chorioallantoic Membrane (HET-CAM) method[5] as a potential alternative to animal experiments. Fertile eggs are widely available, and the egg hatching temperature (37-38 °C) easy to achieve- these makes the HET-CAM a nice alternative experiment platform compared to the animal models. The vascular network formation of the chorioallantoic membrane (CAM) is suitable for studying tumor growth, drugs delivery, tissue xenograft, wound healing, as well as toxicological study.[6] It was reported that the CAM is not innervated. Additionally, the chick embryo advances to a functional brain on EDD 13.[7] Thus, we can consider the HET-CAM model as a humane alternative of animal testing. After adding the materials to the CAM, following three category of irritation reactions can be detected on the CAM: hemorrhage, vascular lysis,

and coagulation. The seconds needed for each type of irritational reaction after applying the materials can be used directly to determine the irritation score (IS) from the following equation.[8]

$$IS = [5\times(301-mth)/300]+[7\times(301-mtl)/300]+[9\times(301-mtc)/300]$$

Here, mth = mean time to haemorrhage (in seconds), mtl = mean time to start vascular lysis (in seconds), mtc = mean time to start coagulation (in seconds). When no irritation component (such as hemorrhage, lysis, or coagulation) was observed within 5 minutes after sample application, the contribution of respective irritation component was considered zero to calculate the total irritation score (IS).

Table S1. Classification of irritant based on the irritation score (IS) [8]

Classification	Irritation Score (IS)
Maximum	21
Severe irritant	> 9
Non-to-moderate irritant	0 - 9

Custom-built confocal microscopy for bioimaging and Intracellular tracking

The confocal setup is a conventional custom-built setup, driven by the software Qudi, which could perform a variety of basic measurement functionalities.

The key hardware of the setup consists of an oil-immersion objective lens (Olympus, 1.35, 60×), a 532 nm continuous-wave laser, a spectrometer (Princeton Instruments, Acton SP

300i), an avalanche photo-diode (APD, Excelitas, SPCM-AQRH), optical filter for confocal imaging is a bandpass of 709 ± 83.5 nm. For compatibility with the ibidi cell-chamber, the recommended ibidi immersion oil by ibidi was used for the objective of the confocal microscope.

On the detection arm, a motorized flip-mirror was used to conduct fluorescence to the APD or the spectrometer, for confocal imaging and tracking, or for spectral measurements respectively. Fast tracking by CCD camera was applied in some of the literatures, which can reveal real-time marker positions on the focal plane in milliseconds. However, there is a sacrifice of losing the position information on the Z-axis. With the positioning excellence of the 3-D piezo scanners on the scanning confocal microscope, as in our case, it takes a few seconds to perform a tracking action but with the advantage that high precision of 3-D trajectories of the cellular marker can be achieved.

Within the fluorescence image, a measurement loop was carried out repeatedly on single bright and photo-stable ND-CP particles. Firstly, a fluorescence scan was performed in a small-range of 100 μm × 100 μm on the X-Y plane, and then zoon in to one single cell and find out the ND-CP fluorescence and measurement the spectrum to prove it is NV center. A 600 nm × 600 nm ×2 μm cube was taken as an optimiser to catch the tracking ND-CP particles. Every 5 second the optimiser was use to find out the best focal point to collect maximum fluorescence counts in the well-aligned scanning confocal setup in the 600 nm × 600 nm ×2 μm. The real-time fluorescence intensity was recorded and the maximum intensity should be kept similar in the whole process. Otherwise the process was given up and to look for other points.

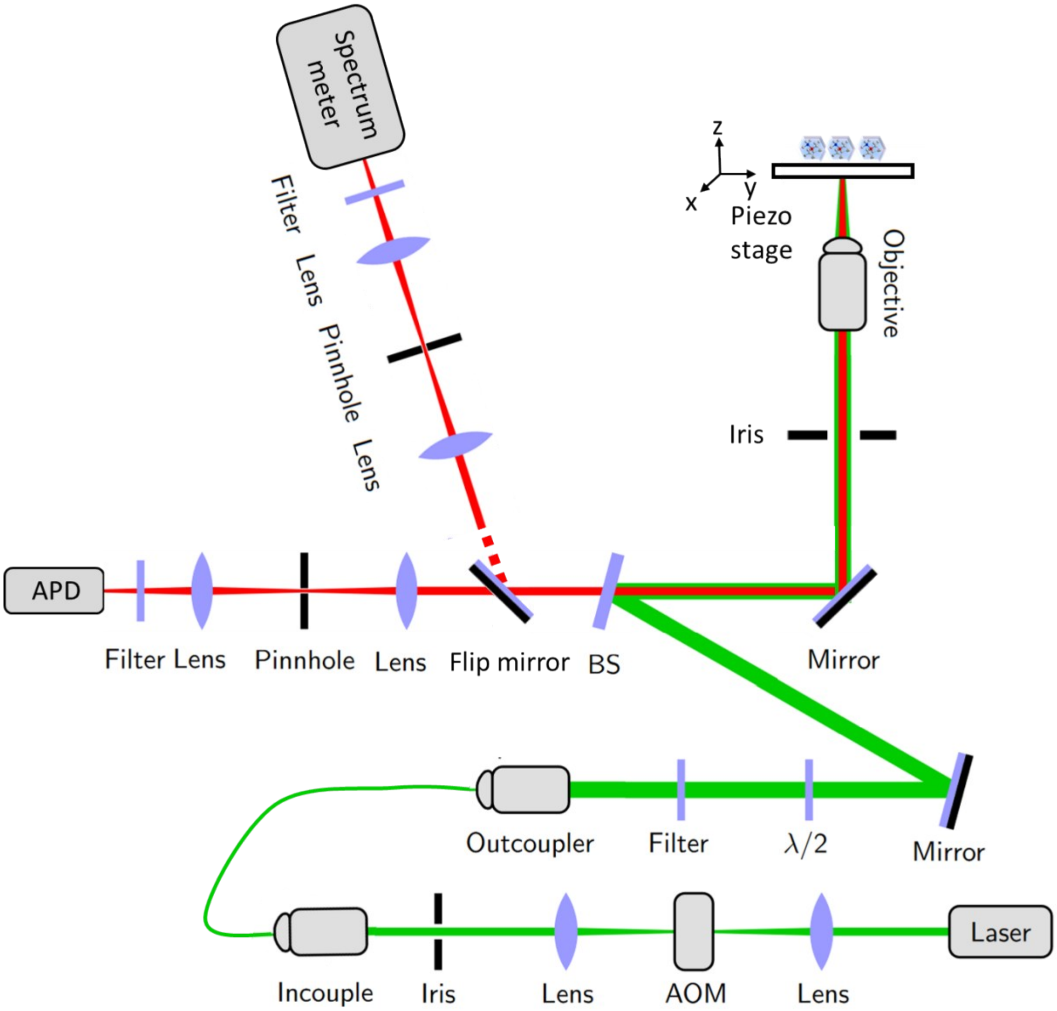

Figure S1. Schematic of the custom-built confocal microscopy

Mean square displacement (MSD) analysis and the calculation of diffusion coefficient (D_0)

The mean square displacement (MSD) data of an intracellular trajectory allows understanding of the diffusion behavior of the tracked object and calculation of the diffusion coefficient. In this work, we recorded three trajectories, labeled as "Trajectory 1", "Trajectory 2", and "Trajectory 3", which contain 520, 88, and 81 position vectors, respectively. Each position vector was recorded every 5 seconds, so the tracking times are about 43 minutes, 7 minutes and 7 minutes for

"Trajectory 1", "Trajectory 2", and "Trajectory 3" individually. The MSDs of the trajectories were calculated as

$$\mathrm{MSD}(t_{\mathrm{lag}}) = \mathrm{MSD}(n\Delta t) = \frac{1}{N-n}\sum_{j=1}^{N-n}\left|\mathbf{r}((n+j)\Delta t) - \mathbf{r}(j\Delta t)\right|^2 , \tag{S1}$$

where t_{lag} is the lag time, Δt is the measurement interval (5 s) of the trajectories, n is the number of intervals contained in the lag time, N is the total number of position vectors in the trajectory, and $\mathbf{r} = [x, y, z]$ is the position vector of the tracked particle. As shown in Figure 4A, the MSDs of the three trajectories show different magnitudes and lag time dependencies. To identify the diffusion behavior of the ND-CP, we fitted the MSD data with a power-law as

$$\mathrm{MSD}(t_{\mathrm{lag}}) = A t_{\mathrm{lag}}^{\alpha} , \tag{S2}$$

where A and α are fitting parameters. In particular the power index α reflects the diffusion behavior of the tracked object, with $\alpha < 1$, $\alpha = 1$, and $\alpha > 1$ indicating confined diffusion, normal diffusion, and directed motion, respectively.[9, 10] According to Saxton[11] et al., we focused on data with $t_{\mathrm{lag}} < t_{\mathrm{total}} / 4$, where t_{total} is the total time of the trajectory. Furthermore, we divided the MSD data into two regions, corresponding to short and intermediate lag times, respectively. Through segmental fitting with Equation S2, we obtained the characteristic power indices of the three trajectories, as indicated in Figure 4A. The power indices are smaller than one at short lag times and become close to one at intermediate lag times, suggesting different diffusion behaviors of the ND-CP at different lag time scales. Similar MSD results have been reported for tracking of single-walled-carbon-nanotube (SWNT)-labeled kinesins in COS-7 cells[12] and the confined diffusion behavior at short lag times could be explained by the existence of mechanical obstacles in the cell[12, 13].

Another way to distinguish normal and anomalous diffusion (either confined diffusion or directed motion) is by calculating the cumulative distribution function (CDF) of the square displacements (Δr^2) at a particular t_{lag}.[9, 14] Here we focused on "Trajectory 1", the longest trajectory recorded in this work, and $t_{\text{lag}} = 2\Delta t = 10$ s. The probability distribution function (PDF) of the square displacements were first calculated (Figure 4B, top panel), based on which the CDF was computed (Figure 4B, bottom panel). We further fitted the CDF data with single and double exponential functions as follows,

$$\mathrm{CDF}(r^2,\ t_{\text{lag}}) = 1 - \exp\left(-r^2/r_0^2\right), \tag{S3}$$

where r_0^2 is the MSD at t_{lag}, and

$$\mathrm{CDF}(r^2,\ t_{\text{lag}}) = 1 - \left[w\exp\left(-r^2/r_1^2\right) + (1-w)\exp\left(-r^2/r_2^2\right)\right], \tag{S4}$$

where r_1^2 and r_2^2 are the MSDs at t_{lag}, corresponding to the fast and slow mobility components, respectively. The contributions of these two components to the CDF are weighted with factors w and (1-w) respectively. Whereas Equation S3 implies normal diffusion, Equation S4 covers both normal (w = 0 or 1) and anomalous ($w \rightarrow 0.5$) diffusion. The r_i^2 (i = 0, 1, 2) are related to the diffusion coefficient D_i as $r_i^2 = 6D_i t_{\text{lag}}$. From the fitting, we obtained $r_0^2 = 1.2540 \times 10^{-2}$ μm^2, $r_1^2 = 1.7715 \times 10^{-3}$ μm^2, $r_2^2 = 1.7590 \times 10^{-2}$ μm^2, and $w = 0.2616$. As a result we obtained the following diffusion coefficients (at t_{lag} = 10 s): $D_0 = 2.09 \times 10^{-4}$ μm^2/s, $D_1 = 2.95 \times 10^{-5}$ μm^2/s, and $D_2 = 2.93 \times 10^{-4}$ μm^2/s. A w value of 0.2616 and the much smaller residual of the double exponential fit than that of the single exponential fit (Figure 4B, top of the bottom panel) confirming that the diffusion behavior of the ND-CP in the HeLa cells is indeed a combination of normal and anomalous diffusion.

We also calculated the lag-time-dependent diffusion coefficient of the ND-CP in HeLa cell according to

$$D(t_{\mathrm{lag}}) = \frac{\mathrm{MSD}(t_{\mathrm{lag}})}{6t_{\mathrm{lag}}}, \quad \text{(S5)}$$

Applying Equation S5 to the three trajectories at intermediate lag times, where the power indices are close to one (Figure 4A), we obtained the following three diffusion coefficients, 1.25×10^{-4}, 3.07×10^{-4}, and 1.39×10^{-3} $\mu m^2/s$ (Figure 4C), which are comparable to the values obtained from the single and double exponential fits to the CDF data at $t_{\mathrm{lag}} = 10$ s. The nominal diffusion coefficient of the ND-CP in the HeLa cell was then determined to be the average of these three values, that is, 6.07×10^{-4} $\mu m^2/s$.

2. Tables and Figures

Table S2. Irritation Score (IS) for different concentrations of ND-CP, compared to 1% SDS (positive control) and PBS (negative control).

**Eggs with sample (n=3)**	**Classification**	**Irritation score (IS)**
1% SDS	Severe irritation	11.36±0.02
PBS	No irritation	0.00
ND-CP (100 µg/mL)	No irritation	0.00
ND-CP (50 µg/mL)	No irritation	0.00

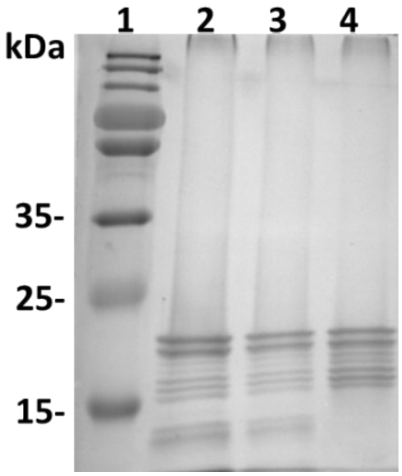

Figure S2. SDS-PAGE images of ND-CP: 1, standard protein ladder; 2, ND-CP in Tris buffer; 3, ND-CP in PBS buffer; 4, CP in Tris buffer. Both lane 2 and 3 present the same band as native CP (MW ≈ 19 kDa) (lane 4), whereas one more band is found with ND-CP, which might be the PVP we added in the ND solution.

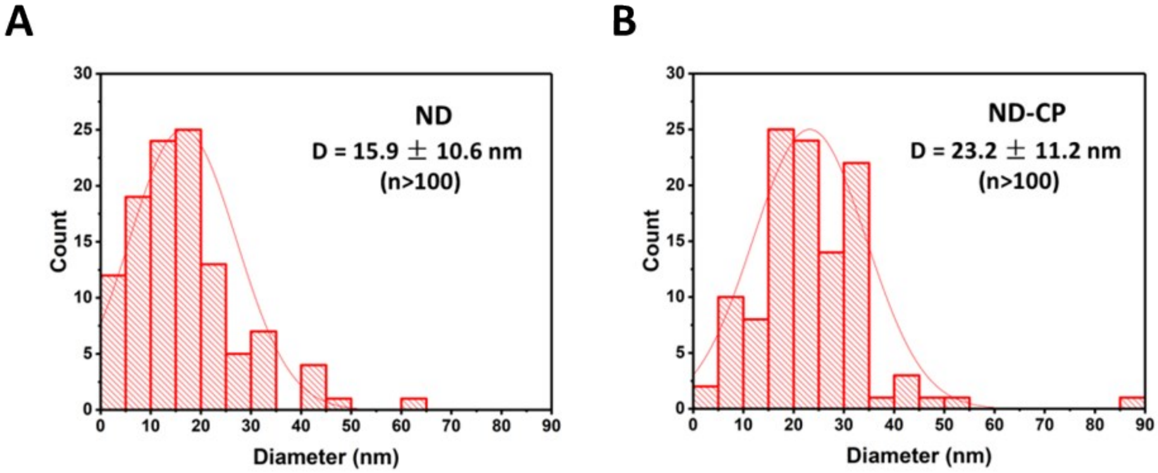

Figure S3. (A) The size distribution histogram of NDs. (B) The size distribution histogram of ND-CPs.

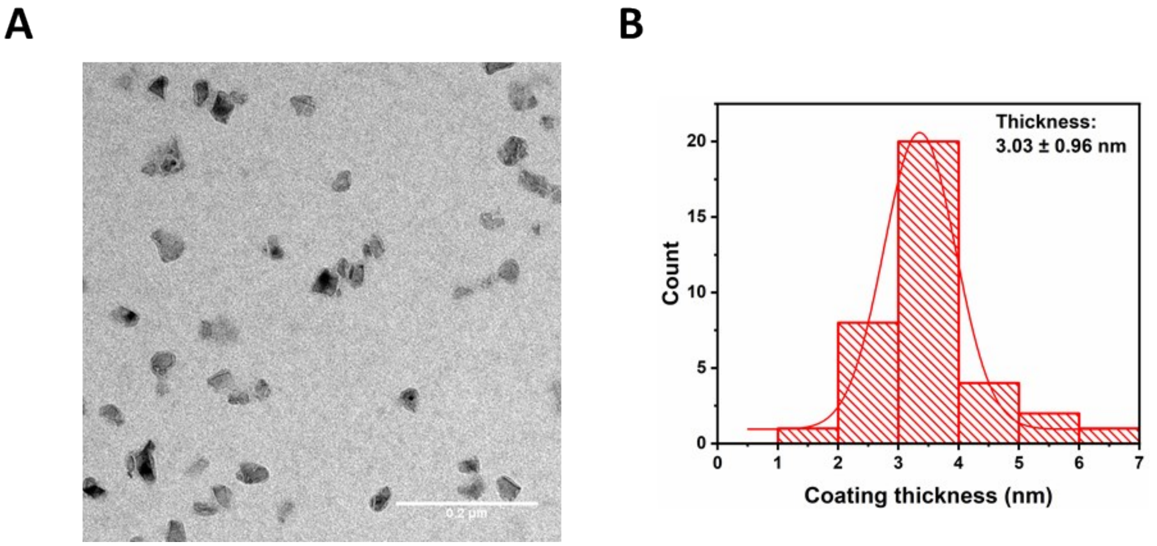

Figure S4. (A) TEM images of ND-CP, negatively stained with 4% uranyl acetate; (B) The shell thickness distribution histogram of ND-CP.

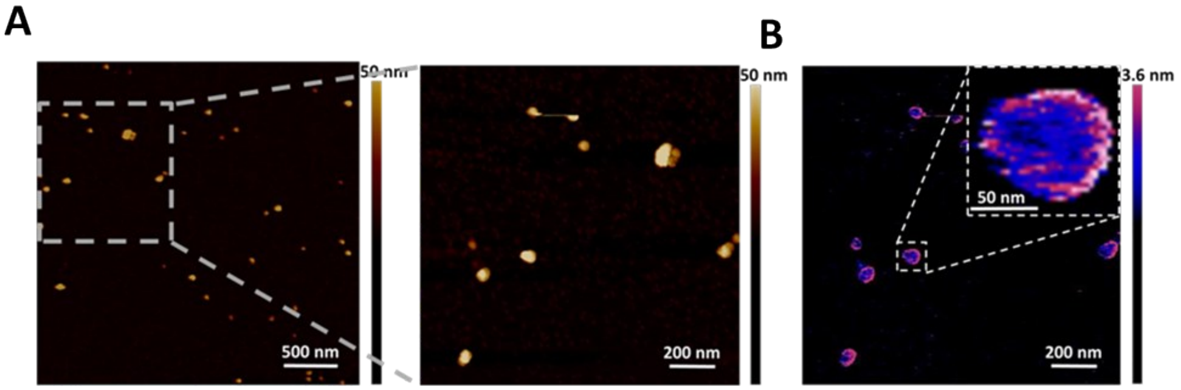

Figure S5. (A) AFM images of ND-CP in liquid state in height sensor; (B) AFM images of ND-CP in deformation sensor.

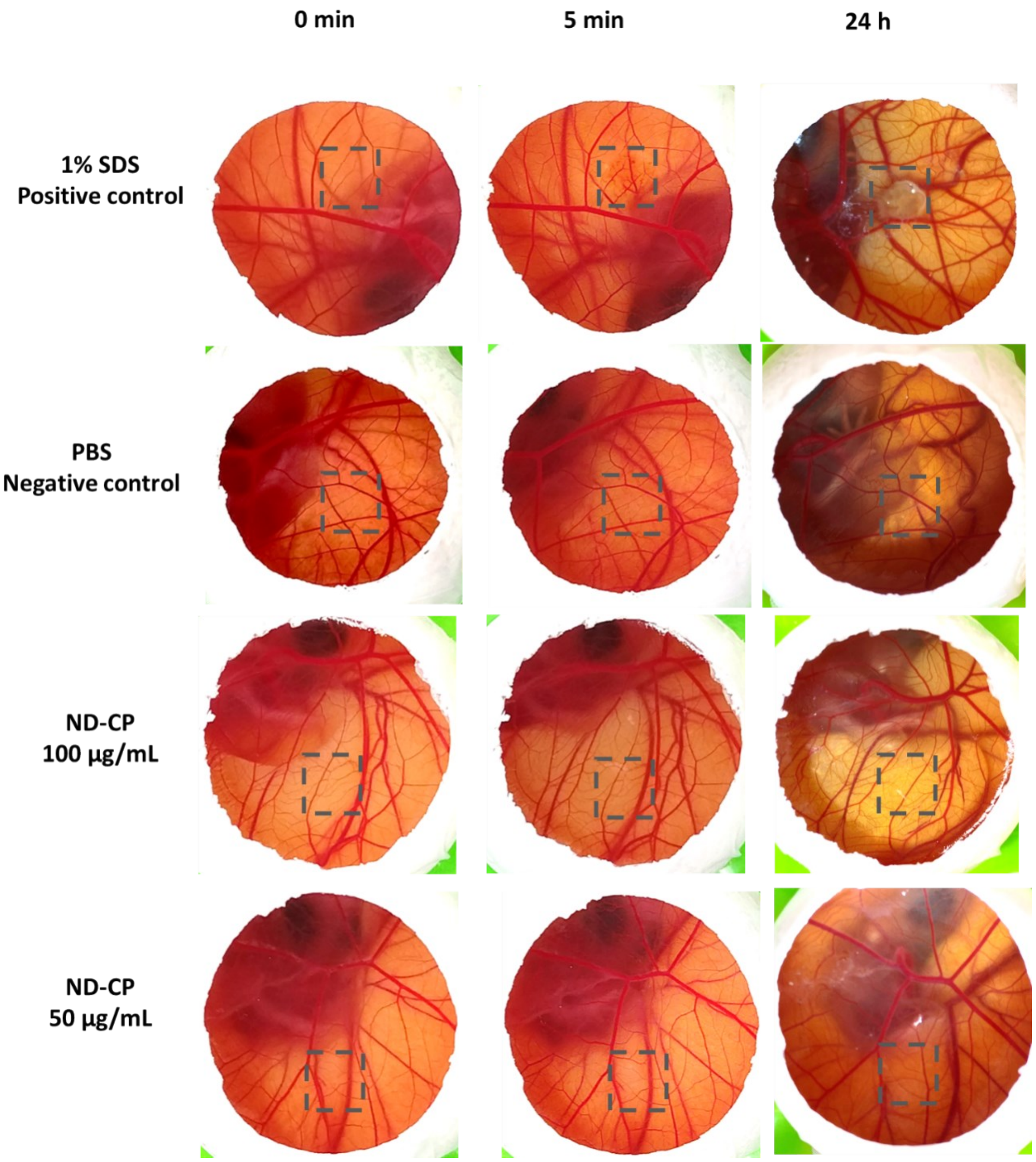

Figure S6. Photographs of HET-CAM test results for 1% SDS (positive control), PBS (negative control) and ND-CP at 50 μg/mL and 100 μg/mL.

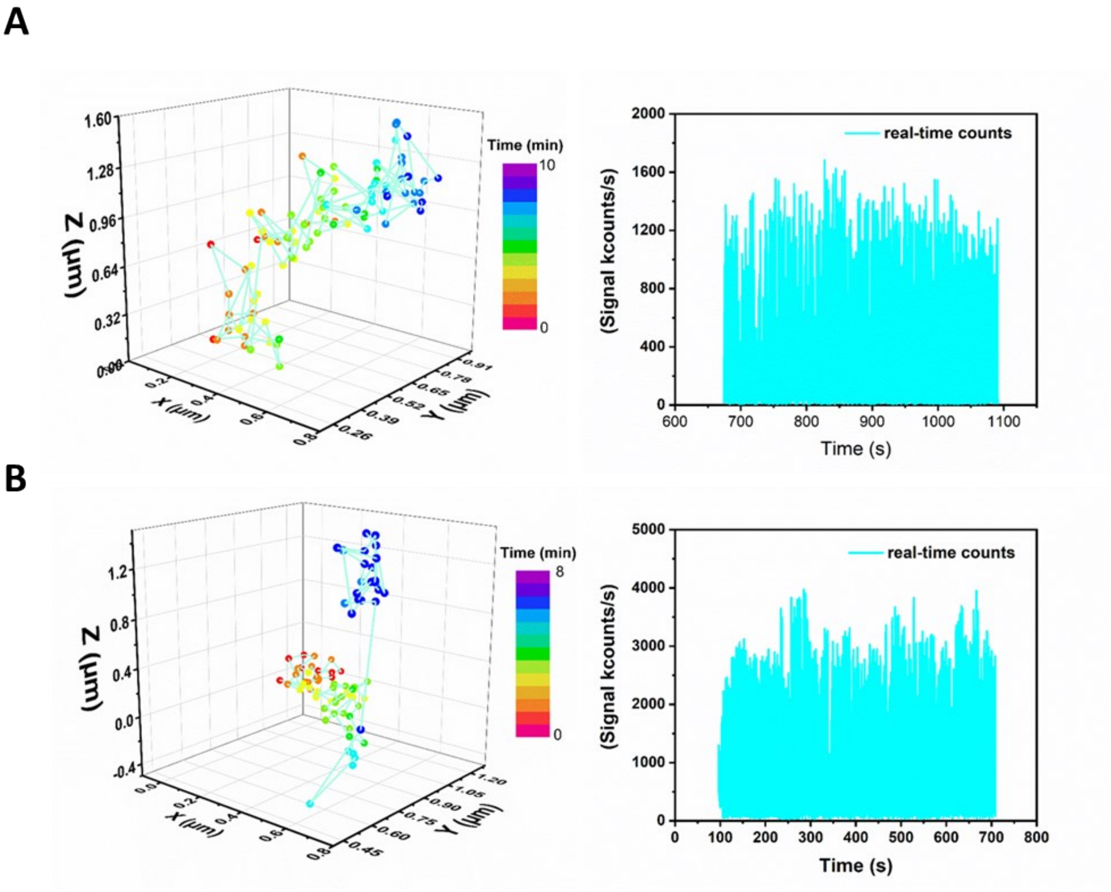

Figure S7. (A) The trajectory of tracked ND-CP spot 2 in intracellular space of HeLa cell and Real-time counts of fluorescence of the tracked ND-CP spot 2; (B) The trajectory of tracked ND-CP spot 3 in intracellular space of HeLa cell and Real-time counts of fluorescence of the tracked ND-CP spot 3.

3. References

1. J. J. Bujarski, in *Plant Virology Protocols*, Springer, 1998, pp. 183-188.
2. B. Verduin, *J. Gen. Virol.*, 1978, **39**, 131-147.
3. R. Hamilton, J. Edwardson, R. Francki, H. Hsu, R. Hull, R. Koenig and R. Milne, *J. Gen. Virol.*, 1981, **54**, 223-241.
4. M. Comellas-Aragonès, H. Engelkamp, V. I. Claessen, N. A. Sommerdijk, A. E. Rowan, P. C. Christianen, J. C. Maan, B. J. Verduin, J. J. Cornelissen and R. J. Nolte, *Nat. Nanotechnol.*, 2007, **2**, 635-639.
5. G. Winter, A. B. Koch, J. Löffler, M. Lindén, C. Solbach, A. Abaei, H. Li, G. Glatting, A.

J. Beer and V. Rasche, *Cancers*, 2020, **12**, 1248.
6. A. Vargas, M. Zeisser-Labouèbe, N. Lange, R. Gurny and F. Delie, *Adv. Drug Delivery Rev.*, 2007, **59**, 1162-1176.
7. E. Aleksandrowicz and I. Herr, *ALTEX-Alternatives to animal experimentation*, 2015, **32**, 143-147.
8. A. Schrage, A. O. Gamer, B. van Ravenzwaay and R. Landsiedel, *Altern. Lab. Anim.*, 2010, **38**, 39-52.
9. A. V. Weigel, B. Simon, M. M. Tamkun and D. Krapf, *Proc. Natl. Acad. Sci. U. S. A.*, 2011, **108**, 6438-6443.
10. N. Gal, D. Lechtman-Goldstein and D. Weihs, *Rheol. Acta*, 2013, **52**, 425-443.
11. M. J. Saxton, *Biophys. J.*, 1997, **72**, 1744-1753.
12. N. Fakhri, A. D. Wessel, C. Willms, M. Pasquali, D. R. Klopfenstein, F. C. MacKintosh and C. F. Schmidt, *Science*, 2014, **344**, 1031-1035.
13. Š. Bálint, I. V. Vilanova, Á. S. Álvarez and M. Lakadamyali, *Proc. Natl. Acad. Sci. U. S. A.*, 2013, **110**, 3375-3380.
14. G. J. Schütz, H. Schindler and T. Schmidt, *Biophys. J.*, 1997, **73**, 1073-1080.
